# Does Podoplanin (PDPN) Reflect the Involvement of the Immunological System in Coronary Artery Disease Risk? A Single-Center Prospective Analysis

**DOI:** 10.3390/ijms262412051

**Published:** 2025-12-15

**Authors:** Tomasz Urbanowicz, Joanna Rupa-Matysek, Ewelina Wojtasińska, Beata Krasińska, Maciej Zieliński, Malwina Grobelna, Paweł Zawadzki, Ryszard Staniszewski, Zbigniew Krasiński, Elżbieta Paszyńska, Andrzej Tykarski

**Affiliations:** 1 Cardiac Surgery and Transplantology Department, Poznan University of Medical Sciences, 61-848 Poznan, Poland; 2 Department of Hematology, Transplantation and Cellular Therapy, Poznan University of Medical Sciences, 60-569 Poznan, Poland; 3 Department of Hypertensiology, Angiology, and Internal Medicine, Poznan University of Medical Sciences, 61-848 Poznan, Poland; 4 Department of Vascular, Endovascular Surgery, Angiology and Phlebology, Poznan University of Medical Sciences, 61-701 Poznan, Poland; 5 Department of Integrated Dentistry and Community Dentistry, Poznan University of Medical Sciences, 60-512 Poznan, Poland

**Keywords:** coronary disease, podoplanin, PDPN, novel markers

## Abstract

Coronary artery disease remains a significant global health challenge, driven by a multifactorial pathophysiology, including immunological activation. The identification and management of potential risk factors are crucial for improving prevention opportunities. In this study, the role of novel, innate immune system response markers, such as podoplanin 38, in atherosclerosis was investigated. A total of 150 consecutive patients (87 (58%) male; median age of 68 (61–76) years) with chronic coronary symptoms (anginal equivalent, e.g., exertional dyspnea) who underwent clinical evaluation and de novo coronary angiography for a prospective single-center analysis were included. Demographic and clinical data, combined with echocardiographic and coronary angiography results, were analyzed in conjunction with laboratory results from admission, including serum podoplanin (PDPN) concentrations. Serum PDPN concentrations were significantly lower in the coronary artery disease group (238 (174–360) pg/mL) compared to the control group (428 (207–1381) pg/mL, *p* = 0.002). A negative correlation was observed between PDPN and the number of involved coronary arteries in the atherosclerotic process (*R* = −0.27, *p* < 0.01). In diabetic populations, glycemic hemoglobin (Hb1Ac) is correlated with the podoplanin concentration (*r* = −0.51, *p* = 0.001). A correlation between PDPN and the left ventricular ejection fraction was noted in both the control (r = 0.33, *p* < 0.01) and CAD groups (r = 0.37, *p* < 0.001). Podoplanin (PDPN) can be considered a novel marker for coronary atherosclerosis. Low serum podoplanin concentrations characterized patients with coronary artery disease.

## 1. Introduction

Podoplanin, a mucin-type transmembrane glycoprotein (PDPN), plays an integral role in regulating immunity development and function, as well as cancerogenesis [1]. It is a sialomucin-like type I protein that has recently been extensively studied due to its expression in various cells and tissues, including lymphatic endothelial cells, alveolar cells, osteocytes, carcinoid cells, and lymphoid organs [2]. Podoplanin’s structure includes a heavily glycosylated extracellular domain, a transmembrane domain, and a short intracellular domain with approximately 130, 25, and 10 amino acids, respectively [3]. Podoplanin primarily functions through protein–protein interactions on three levels: extracellular, transmembrane, and intracellular. The proteins may bind to either the extracellular domain via C-type lectin-like receptor-2 (CLEC-2) (described in stroke, brain injury, and tumors), heat shock protein A9 (HSPA9) (postulated in oncological disease including metastatic spread), or chemokine ligands (CCL21) (in cancer invasion) [4,5,6,7]. The PDPN extracellular domain may interact with the lymphatic endothelium and extracellular matrix through galectin [8,9,10]. Adaptive immune activation and cancerous disease progression are linked to the interplay between the transmembrane domain and cell surface proteins CD9 and CD44 [11,12]. The role of the intracellular domain in the epithelial–mesenchymal transition (EMT) in cancer cells is linked to ERM [13].

PDPN acts as an endogenous ligand of the platelet activation receptor (CLEC-2), which is essential for maintaining hemostasis and vascular integrity [14].

Coronary artery disease remains a major global health challenge, driven by a multifactorial pathophysiology. The identification and management of potential risk factors are crucial for enhancing prevention opportunities [15]. Novel analyses have identified metabolomic profiling as a potential indicator of coronary artery disease risk and a future therapeutic target [16,17,18]. The literature has established atherosclerosis’ underlying pathophysiological paradigm related to plaque formation not merely as a result of dyslipidemia and the arterial hypertension interplay but as a chronic, immune-mediated inflammatory disorder.

PDPN upregulation was observed in immunohistochemical analyses of atherosclerotic lesions [19]. In acute phages, PDPN is implicated in thrombus formation on disrupted plaques by triggering platelet activation through the platelet C-type lectin-like receptor 2 (CLEC-2) and the Src–Syk–SLP-76 signaling pathway [20]. The PDPN expression is induced locally in atherosclerotic lesions, facilitating platelet–cell interactions that result in both immune activation and increased thrombotic risk [20]. The PDPN can be regarded as a potential marker of atherosclerosis-related thromboinflammatory crosstalk.

The involvement of the inflammatory system in atherosclerotic plaque development and progression has led to a focus on novel markers and potential future therapies [21,22,23]. Novel markers related to the immune system and prothrombotic state activation have emerged as pivotal diagnostic and prognostic tools for assessing atherosclerosis. The initiation and progression of atherosclerotic plaques involve a complex interplay between modified lipoproteins, activated endothelial cells, monocyte-derived macrophages, and T-lymphocytes, which collectively orchestrate a sustained inflammatory response. As the inflammatory and prothrombotic background of coronary atherosclerosis has been postulated, recent studies have noted the potential modulatory effect of statin therapy [24,25,26].

One of the potential novel markers of atherosclerosis is podoplanin, indicating a possible link between plaque formation and the immune system. The expression of this transmembrane glycoprotein (PDPN) has been observed in immune cell populations, podocytes, lymphatic endothelial cells, and other cell types. PDPN is traditionally associated with lymphangiogenesis processes and tumor metastasis. In animal models, Ref. [14] PDPN deficiency resulted in cerebrovascular abnormalities. 

This study aimed to compare possible differences in the serum podoplanin concentration between patients with angiographically proven de novo coronary artery disease and a control group (patients with normal coronary angiograms) to assess its potential as a CAD biomarker.

## 2. Results

**Figure 1:**
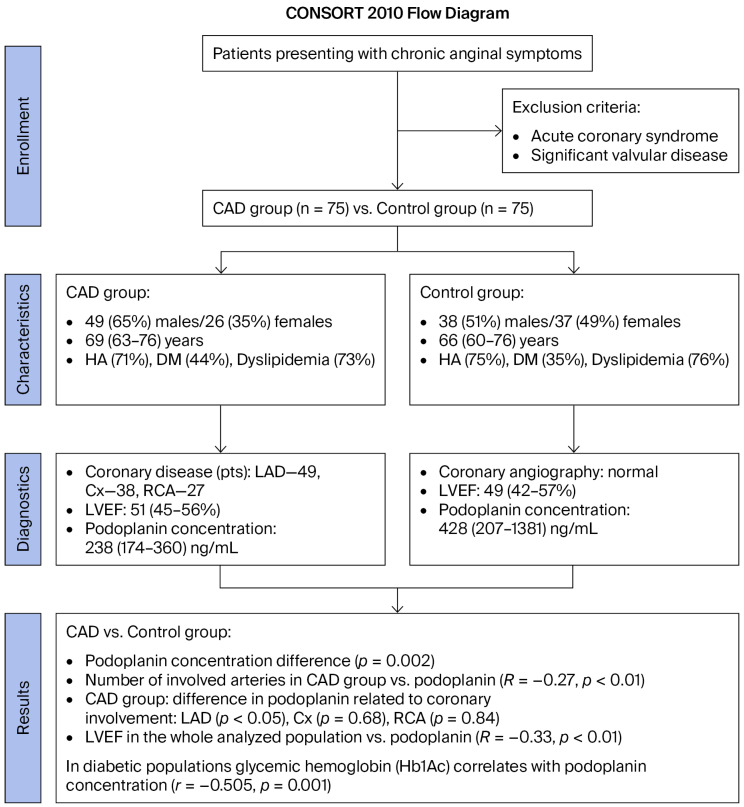
Flow chart. Abbreviations: CAD—coronary artery disease, Cx—circumflex artery, DM—diabetes mellitus, HA—arterial hypertension, LAD—left descending artery, LVEF—left ventricular ejection fraction, and RCA—right coronary artery.

We enrolled 150 consecutive patients (87 (58%) male; median age of 68 (61–76) years) with chronic coronary symptoms (anginal equivalent, e.g., exertional dyspnea) who underwent a clinical evaluation and de novo coronary angiography for a prospective single-center analysis (Figure 1).

Seventy-five patients (49 (65%) male; median age 69 (63–76) years) with angiographically confirmed CAD (CAD group) were compared to seventy-five patients (38 (51%) male; median age 66 (60–76) years) with normal coronary arteries (control group). No significant differences were identified between the groups’ sex (*p* = 0.07), age (*p* = 0.67), body mass index (*p* = 0.42), or co-morbidities, including arterial hypertension (*p* = 0.58), dyslipidemia (*p* = 0.49), or diabetes mellitus (*p* = 0.09) (Table 1). There were no differences in pharmacotherapy between the two groups, as presented in Table 1.

Serum podoplanin concentrations (Pg38) were significantly lower in the CAD group (238 (174–360) pg/mL) compared to the control group (428 (207–1381) pg/mL, *p* = 0.002). Other laboratory parameters, including CBC, glycemic hemoglobin, lipid profiles, and kidney and liver function tests, did not differ between groups (Table 2).

The PDPN concentration did not differ significantly across the entire analyzed population regarding sex (*p* = 0.134), arterial hypertension (*p* = 0.299), or dyslipidemia (*p* = 0.397). There was no correlation between serum PDPN levels and age (r = 0.04, *p* = 0.791).

Echocardiography parameters, including the left ventricular ejection fraction (LVEF) (*p* = 0.55), left ventricular diastolic diameter (*p* = 0.71), left atrial diameter (*p* = 0.88), and interventricular septum thickness (*p* = 0.013), were similar between groups (Table 2). Positive correlations were observed between the LVEF and podoplanin levels in both the control group (r = 0.33, *p* < 0.01) and the CAD group (r = 0.37, *p* < 0.001).

We identified a negative correlation between the PDPN serum concentration and the number of arteries involved in the atherosclerotic process (*R* = −0.27, *p* < 0.01) in the CAD group, as well as a correlation with glycemic hemoglobin in the diabetic population (*R* = −0.505, *p* = 0.001).

## 3. Discussion

This prospective study suggests that lower levels of serum podoplanin (PDPN) concentrations are associated with an increased risk of coronary artery disease (CAD) and may serve as a novel biomarker. In a previous experimental study [27], PDPN deletion increased inflammatory signaling in lymph nodes and disrupted immune responses. 

The role of platelets in atherosclerosis has been postulated; however, the underlying mechanisms still require investigation, as they play a significant role in both innate and adaptive immunity [28]. Podoplanin is considered an endogenous ligand of the platelet activation receptor. C-type lectin-like receptor-2 (CLEC-2), expressed on the surface of platelets, belongs to the C-type lectin superfamily and binds to podoplanin, inducing its activation and aggregation [29]. In animal models [30], the deletion of the CLEC-2 receptor (located in the podoplanin extracellular domain) results in reduced platelet accumulation in the subendothelium. In animal models, podoplanin neutralization has had a significant impact on acute cardiovascular events, including stroke and infarct sizes [31,32]. On the other hand, our results, focused on the chronic state, suggest a possible link between lower podoplanin levels and coronary atherosclerosis, which may be explained by several possible mechanisms. Podoplanin is bound to the CLEC-2 receptor on platelets and the surface of immune cells, resulting in the sequestration of PDPN and a decrease in the circulating levels of free podoplanin [33]. Consumption and sequestration could provide an explanation for these results. A reduced lymphatic and endothelial repair capacity may be another factor responsible for lower circulating pg38 levels. As a role of podoplanin in lymphatic vessel formation and endothelial maintenance has been postulated, a low podoplanin concentration may indicate impaired lymphoangiogenesis, inadequate vascular repair, and impaired endothelial maintenance.

Differences in PDPN, as presented, were observed between the analyzed groups, despite the use of the antiplatelet therapy in the entire enrolled population. Our results suggest a novel, independent mechanism from antiplatelet action, highlighting a possible role of platelets and their receptors in the functions of PDPN in coronary artery disease.

PDPN is a cell surface mucin-like glycoprotein involved in the development of heart and lymphatic vascular systems [34]. This transmembrane protein, by binding to specific proteins, regulates various cellular events, including the epithelial–mesenchymal transition and extracellular matrix remodeling [11]. Its role in mammary stem cell activity and platelet biogenesis is postulated, indicating a potential role in immune responses [6]. Podoplanin expression is upregulated in various cell types during inflammation, including fibroblasts, macrophages, T helper cells, and epithelial cells [35]. Lower PDPN levels in the patients presenting with chronic coronary syndrome compared to the control group may indicate the loss of protective anti-inflammatory signaling. Podoplanin plays a protective role by modulating macrophage activation, vascular remodeling, and homeostasis, thereby favoring plaque formation [36].

The negative correlation between podoplanin levels and the number of diseased coronary arteries, as well as glycemic hemoglobin (HbA1c), in diabetic patients aligns with PDPN’s potential role in the pathophysiology of CAD. Our analysis suggests that poor diabetes control, measured by Hb1Ac, predisposes individuals to lower PDPN levels. Jakubiak et al. [37] discussed the relationships between HbA1c and the risk of coronary heart disease in their review. In contrast, the OPTIMAL randomized clinical trial results did not support the association between optimal glucose control and CAD progression in diabetic patients [38].

Additionally, podoplanin’s involvement in vascular remodeling suggests a broader role in the progression of vascular diseases beyond coronary atherosclerosis. PDPN overexpression has been investigated in several tumors and linked with their malignancy properties. This glycoprotein induces platelet aggregation; modulates signal transduction; and regulates cell proliferation, differentiation, migration, and invasion [39].

PDPN’s involvement in lymphoangiogenic processes in autoimmune diseases is associated with clinical severity and lymphatic vessel density [40]. The interplay between cardiovascular morbidity and the structure of the lymphatic vasculature has been expertly reviewed [41]. According to a previous report [42], the pathological remodeling of lymphatic vessels, including their aberrant function, may impact coronary atherosclerosis more than their quantity, which may determine their role in plaque formation.

This study highlights a novel association between lower serum podoplanin concentrations and an increased risk of CAD, suggesting a complex pathophysiology. Podoplanin’s role as a marker of lymphoangiogenesis, inflammation, and platelet activation [43] suggests that reduced PDPN levels in patients with angiographically confirmed atherosclerosis may reflect endothelial dysfunction and impaired vascular remodeling, including lymphoangiogenesis. Thus, PDPN is crucial for the development and function of lymphatic vessels. This may lead to reduced podoplanin expression in CAD, suggesting its potential role in the pathomechanism of this disease. Our analysis investigated serum PDPN levels in coronary atherosclerosis, in contrast to previous histopathologic reports that revealed PDPN in aortic plaques [19].

Podoplanin is considered an endogenous ligand of the platelet activation receptor. Platelet hyperreactivity is considered a key factor in atherosclerosis formation, as platelets bind native low-density lipoprotein (nLDL) and oxidized LDL (oxLDL) via a platelet-specific LDL receptor (LDLR), ApoE-R2 [44]. Platelets, in turn, can modulate lipoprotein metabolism by releasing platelet factor 4 and transforming growth factor β, which in turn modulates LDL uptake and foam cell formation. The presented results may suggest possible platelet dysfunction in CAD patients, independent of antiplatelet therapy. This finding is novel and intriguing, although further investigation is required to confirm its implications.

In chronic coronary syndrome, dysregulated inflammatory responses, particularly in advanced disease stages, may lead to a reduction in the expression of podoplanin (PDPN) in podoplanin-expressing cells. In our analysis, we observed decreased PDPN serum concentrations, potentially due to chronic ischemia that causes a loss of podoplanin-expressing myocardial and endothelial cells secondary to coronary atherosclerosis.

The role of PDPN in diabetes has been investigated as it contributes to diabetic kidney disease and islet fibrosis. Podoplanin expressed in podocytes is critical for maintaining the glomerular filtration barrier. Hyperglycemia and oxidative stress can reduce its expression, leading to podocyte injury and detachment, which in turn can cause proteinuria. In previous reports [45,46,47], high glucose levels were found to decrease podoplanin levels, leading to kidney dysfunction and exacerbating inflammation. In diabetes, poor tissue regeneration and chronic edema are associated with chronic inflammation and lymphatic dysfunction, which result from the decreased expression of podoplanin. Additionally, podoplanin expression may alter platelet function in diabetes, leading to an increased risk of microvascular thrombosis. In our analysis of diabetic patients, lower podoplanin levels were associated with diabetic nephropathy. Glycated hemoglobin is associated with diabetes, reflecting the effectiveness of diabetes control, and its high levels have been linked to increased inflammation [48]. There are various mechanisms that may activate responses related to uncontrolled diabetes through advanced glycation end-products and hemoglobin–heptoglobin complexes [49,50]. In our study, an inverse correlation was noted between gp38 and glycemic hemoglobin in diabetic patients. The proatherogenic impact of poorly controlled diabetes has been previously reported [51]. These results suggest that podoplanin may be a promising biomarker for CAD, particularly in patients with diabetes.

Finally, when analyzing soluble podoplanin (PDPN), one should not consider its serum concentration as a simple, linear readout of the tissue PDPN expression. Circulating PDPN is the result of at least four distinct biological processes. This glycoprotein is released from the cell surface through regulated ectodomain shedding, carried out by ADAM family proteases and matrix metalloproteinases (MMPs) [52]. The susceptibility of a given membrane protein to sheddases depends on its stalk/stem sequence and its post-translational modifications, as presented in an experimental study [53]. The third mechanism may be related to PDPN sequestration by high-affinity receptors (most notably CLEC-2) on platelets and certain leukocytes [54]. The circulating concentration of PDPN may contain soluble ectodomain as a result of clearance and degradation, including hepatic uptake, proteolytic trimming, renal filtration, or capture by scavenger receptors [55].

### Study Limitations

This single-center prospective study included consecutive patients with anginal equivalent symptoms, with CAD defined solely by coronary angiography without functional tests.

Active tobacco users were excluded from the analysis, as previous reports suggested increased PDPN levels in this population [55]. To obtain more objective results, we eliminated patients with one of the three CAD risk factors.

Due to the limited number of participants, a multivariable analysis was not performed. A large-volume, multicenter study including microvascular disease assessments is required to confirm the presented hypothesis.

## 4. Materials and Methods

A prospective single-center analysis enrolled 150 consecutive patients (with chronic coronary symptoms (anginal equivalent, e.g., exertional dyspnea)) who underwent primary clinical evaluation and coronary angiography between 2024 and 2025 in the Internal Disease and Arterial Hypertension Department in Poznan, Poland. Exclusion criteria included acute coronary syndrome, significant valve pathology, current hematological/oncological disease, or nicotine smoking. Demographic, clinical, pharmacological, and laboratory data, including podoplanin serum concentration, were collected on admission. Normal coronary arteries were defined as having less than 20% lumen narrowing on coronary angiograms, in contrast to coronary artery disease, which was defined as more than 50% lumen reduction, assessed by an experienced interventional cardiologist in the reference hemodynamic center. Aggressive statin therapy was defined as daily doses of ≥20 mg of rosuvastatin and ≥40 mg of atorvastatin.

This study was approved by the Institutional Ethics Committee of Poznan University of Medical Sciences (protocol code 113/21, 6 November 2021). Informed consent was obtained from all participants.

### 4.1. Biochemical Analysis

To quantify the human podoplanin (PDPN) concentration in serum, the Human Podoplanin ELISA Kit (Cat. No. 201-12-0124, Shanghai Sunred Biological Technology Company Ltd., Shanghai, China) was used. Study wells were pre-coated with human monoclonal anti-podoplanin antibodies. Both calibrators and patient samples were simultaneously incubated with a secondary anti-PDPN antibody labeled with biotin, followed by the addition of streptavidin–HRP to form an immune complex. After incubation and washing, unbound enzymes were removed. The substrate, tetramethylbenzidine (TMB), was added to the solid phase. An acid-stopping solution was then added to halt the reaction and convert the color from blue to yellow. The intensity of the yellow color was measured using a spectrophotometer at 450 nm. The absorbance was directly proportional to the concentration of human podoplanin in the sample. A dose–response curve of the absorbance versus concentration was generated using calibrator results. Human podoplanin concentrations were determined directly from this curve.

### 4.2. Statistical Analysis

Statistical analysis was performed using JASP (Version 0.14.1, University of Amsterdam, The Netherlands, 2020). Significance was set at *p* < 0.05. The normality of the variable distributions was tested using the Shapiro–Wilk test. The *t*-test and Mann–Whitney test were used to describe and compare the variables between the two groups, when applicable. Spearman tests were used to reveal possible correlations.

## 5. Conclusions

Low serum podoplanin concentrations are associated with an increased risk of coronary artery disease. This glycoprotein may be regarded as a potential novel biomarker for CAD, describing the non-classical pathophysiology of coronary atherosclerosis related to dysfunctional immunological and endothelial activation; however, further studies are required to confirm its clinical utility.

## Figures and Tables

**Table 1:** Demographic and clinical characteristics of the analyzed groups.

Parameters	CAD Group n = 75	Control Group n = 75	*p*
Demographic			
Age (years, median (Q1–Q3))	69 (63–76)	66 (60–76)	0.67
Sex (male (%)/female (%))	49 (65)/26 (35)	38 (51)/37 (49)	0.07
BMI (kg/m^2^) (median (Q1–Q3))	29.1 (26.2–31.2)	28.7 (26.8–33.7)	0.42
Co-morbidities			
Arterial hypertension (n (%))	53 (71)	56 (75)	0.58
Diabetes mellitus (n (%))	33 (44)	26 (35)	0.09
Dyslipidemia (n (%))	55 (73)	57 (76)	0.49
Peripheral artery disease (n (%))	17 (23)	12 (16)	0.41
COPD (n (%))	5 (7)	8 (11)	0.56
Kidney dysfunction * (n (%))	7 (9)	3 (4)	0.20
Atrial fibrillation (n (%))	7 (9)	11 (15)	0.53
Pharmacotherapy			
B-blockers (n (%))	73 (97)	72 (96)	1.00
ACE-I (n (%))	43 (57)	45 (60)	0.87
ARB (n (%))	10 (13)	11 (15)	1.00
CCB (n (%))	14 (19)	17 (23)	0.68
Diuretics (n (%))	23 (31)	21 (28)	0.86
Aspirin (n (%))	75 (100)	75 (100)	1.00
NOAC (n (%))	7 (9)	11 (15)	0.45
SGLT2i (n (%))	13 (17)	12 (16)	1.00
Statins (n (%))	55 (73)	57 (76)	0.85
High-dose statin therapy (n (%))			
Esetimibe (n (%))	11 (15)	10 (13)	1.00
Metformin (n (%))	33 (44)	26 (35)	0.32

Abbreviations: ACE-I—angiotensin convertase enzyme inhibitor, ARB—angiotensin receptor blocker, BMI—body mass index, CAD—coronary artery disease, CCB—calcium channel blocker, COPD—chronic obstructive pulmonary disease, n—number, NOAC—novel oral anticoagulants, SGLt2i—sodium–glucose cotransporter 2 inhibitor, and Q—quartile. * GFR below 60 mL/min/1.73 m^2^.

**Table 2:** Laboratory, echocardiographic, and coronary angiography results.

Parameters	CAD Group n = 75	Control Group n = 75	*p*
Laboratory results (median (Q1–Q3))			
1. Peripheral blood analysis			
White blood count (×10^9^/dL)	7.2 (5.9–7.9)	7.0 (6.0–8.0)	1.00
Hemoglobin (mmol/dL)	9.0 (8.3–9.6)	8.7 (8.1–9.3)	0.45
Platelets (×10^9^/dL)	241 (209–272)	227 (187–288)	0.74
2. Kidney function tests			
Glomerular filtration rate (mL/min/1.73 m^2^)	65 (63–67)	68 (62–77)	0.16
3. Liver function tests			
Alanine transaminase (IU/dL)	25 (19–34)	29 (19–44)	0.46
4. Glycemic hemoglobin (Hb1Ac) (%)	6.5 (5.9–7.0)	6.1 (5.3–6.5)	0.09
5. Lipidogram (mmol/L)			
Total cholesterol	153 (124–183)	153 (123–181)	0.92
High-density lipoprotein	46 (39–58)	55 (44–74)	0.08
Low-density lipoprotein	73 (53–123)	74 (50–111)	0.84
Triglycerides	87 (63–173)	76 (48–101)	0.08
PDPN serum concentration (ng/mL)	238 (174–360)	428 (207–1381)	0.002
Echocardiography on admission			
Left ventricular diastolic diameter (mm) (median (Q1–Q3))	51 (45–56)	49 (42–57)	0.71
Left atrium diameter (mm) (median (Q1–Q3))	38 (36–41)	38 (35–45)	0.88
Interventricular septum (mm) (median (Q1–Q3))	11 (10–12)	12 (11–13)	0.013
Mitral regurgitation (grade) (mean (SD))	1.2 (0.4)	1.2 (0.4)	0.47
Tricuspid regurgitation (grade) (mean (SD))	1.1 (0.4)	1.3 (0.5)	0.88
Left ventricular ejection fraction (%) (median (Q1–Q3))	56 (46–60)	57 (48–62)	0.55
Coronary angiography results (n (%))			<0.001
1—vessel disease	25 (33)	0 (0)	
2—vessel disease	25 (33)	0 (0)	
3—vessel disease	25 (33)	0 (0)	

Abbreviations: dL—deciliter, Hb1Ac—glycemic hemoglobin, IU—unit, L—liter, mL—milliliter, min—minute, mm—millimeter, m^2^—square meter, mmol—millimole, n—number, ng—nanogram, SD—standard deviation, and Q—quartile.

## Data Availability

The data presented in this study are available on request from the corresponding author.
